# Improved bioethanol productivity through gas flow rate-driven self-cycling fermentation

**DOI:** 10.1186/s13068-020-1658-6

**Published:** 2020-01-24

**Authors:** Jie Wang, Michael Chae, David C. Bressler, Dominic Sauvageau

**Affiliations:** 1 Department of Agricultural, Food and Nutritional Science, University of Alberta, Edmonton, T6G 2P5 Canada; 2 Department of Chemical and Materials Engineering, University of Alberta, Edmonton, T6G 1H9 Canada

**Keywords:** Cellulosic ethanol, Batch fermentation, Self-cycling fermentation, Online monitoring parameter, Gas flow rate, Ergosterol and Tween 80, Anaerobic fermentation, Ethanol volumetric productivity, Annual ethanol productivity, Flocculation

## Abstract

**Background:**

The growth of the cellulosic ethanol industry is currently impeded by high production costs. One possible solution is to improve the performance of fermentation itself, which has great potential to improve the economics of the entire production process. Here, we demonstrated significantly improved productivity through application of an advanced fermentation approach, named self-cycling fermentation (SCF), for cellulosic ethanol production.

**Results:**

The flow rate of outlet gas from the fermenter was used as a real-time monitoring parameter to drive the cycling of the ethanol fermentation process. Then, long-term operation of SCF under anaerobic conditions was improved by the addition of ergosterol and fatty acids, which stabilized operation and reduced fermentation time. Finally, an automated SCF system was successfully operated for 21 cycles, with robust behavior and stable ethanol production. SCF maintained similar ethanol titers to batch operation while significantly reducing fermentation and down times. This led to significant improvements in ethanol volumetric productivity (the amount of ethanol produced by a cycle per working volume per cycle time)—ranging from 37.5 to 75.3%, depending on the cycle number, and in annual ethanol productivity (the amount of ethanol that can be produced each year at large scale)—reaching 75.8 ± 2.9%. Improved flocculation, with potential advantages for biomass removal and reduction in downstream costs, was also observed.

**Conclusion:**

Our successful demonstration of SCF could help reduce production costs for the cellulosic ethanol industry through improved productivity and automated operation.

## Background

The US Energy Independence and Security Act of 2007 established a mandatory goal of producing 16 billion gallons of biofuel from lignocellulosic materials by 2022 [1], with cellulosic ethanol being the primary commodity. As a result, big breakthroughs in biomass conversion have been made. For example, POET-DSM claimed that a bottleneck in their pretreatment technology was resolved by enhanced enzymatic digestion of feedstocks [2], the National Renewable Energy Laboratory found that CelA cellulase from *Caldicellulosiruptor bescii* could efficiently hydrolyze cellulose with a high degree of crystallinity [3], and Nguyen et al. demonstrated a great improvement of ethanol titer (86 g/L) and low enzyme dosage (~ 6.5 filter paper unit g glucan^−1^) by combining a cosolvent-enhanced lignocellulosic fractionation pretreatment strategy with simultaneous saccharification and fermentation [4]. As such, while well-recognized technical barriers to viable commercialization of cellulosic ethanol are overcome, the fermentation process itself is now identified as a limiting factor; with current techniques limited by relatively low productivity and intensive labor. Therefore, it is strategically important to develop advanced processes and pursue improvements in productivity for fermentation itself.

We previously reported on a proof-of-concept study showing how a manual cycling fermentation approach used for *Saccharomyces cerevisiae* significantly improved ethanol volumetric productivity and annual ethanol productivity—by 43.1 ± 11.6% and 33.1 ± 7.2%, respectively—compared to batch operation [5]. Self-cycling fermentation (SCF) is an automated semi-continuous fermentation technique in which the onset of stationary phase, identified in real-time by a monitoring parameter, such as dissolved oxygen or carbon dioxide evolution rate (CER) in aerobic systems [6–9], triggers a cycling process. At this point, half the volume of the culture is harvested and immediately replaced by fresh medium to start the next cycle. The repetition can proceed for a number of cycles, e.g., 137 cycles for the production of citric acid [7], without contamination. During SCF operation an elevated degree of cell synchrony, where a large number of cells inside the reactor are at the same phase in their life cycle, has often been achieved with various microbial populations [7, 9, 10]. More details and principles regarding the concept of SCF have been described elsewhere [8, 9, 11]. The proof-of-concept study was carried out in 500-mL shake flasks where SCF was manually mimicked for a total of five cycles [5]. On top of that, the present study is focused on how to implement real SCF into ethanol production under anaerobic conditions, how to automate the process in a 5-L fermenter, and finally, whether the real SCF process can help improve productivity.

To adapt SCF to ethanol fermentation, it is necessary to select a monitoring parameter that clearly identifies the onset of stationary phase in real-time to initiate the automated cycling process. Despite the fact that SCF has been applied to many microbial fermentation systems, the majority of them were operated under aerobic conditions [7–9, 11]. In fact, only two research groups examined the operation of SCF under anaerobic conditions [12–14]—investigating microbial degradation of nitrate species in the former and ethanol production by yeast in the latter. In both cases, the researchers attempted to use oxidation–reduction potential as a monitoring parameter. Unfortunately, their work demonstrated that this parameter was not appropriate to establish reliable cycling processes.

Ethanol fermentations operated in batch mode have also been studied using online monitoring approaches, as a way to reduce the intensive labor necessary for offline chemical analysis [15]. For instance, near-infrared [16], Fourier transform infrared [17], and Raman spectroscopies [18] have all been used and mathematically correlated with concentrations of sugars and ethanol or microbial biomass to indicate the status of fermentations. These strategies are promising and could be adapted as monitoring parameters in SCF. However, the possibility of spectroscopic signals being masked or interfered by the presence of solids in the bioreactor [16, 18] could be of concern, as is the need to build spectral libraries by calibrating with a large number of samples from various conditions [16, 17]. Alternatively, as a co-product of ethanol generation and a proxy for metabolic activity, the CO_2_ produced during fermentation could provide a strategy for operation of SCF with *S. cerevisiae* under anaerobic conditions. Sablayrolles et al. [19] measured the weight loss of a bioreactor, which was presumed to result primarily from the release of CO_2_, as an indicator for fermentation speed in batch mode. However, weighing reactors introduces potentially insurmountable logistical challenges at larger scales.

In the present study, we initially performed batch fermentation of *S. cerevisiae* to monitor patterns in flow rate of outlet gas released—measured using a mass flow meter—as a reliable monitoring parameter to be used for the feedback control of SCF operation. We then incorporated this strategy into an automated SCF system operated under anaerobic conditions. The necessity of adding ergosterol and Tween 80 to reduce fermentation time and improve stability of SCF was also assessed under anaerobic conditions. Finally, an automated SCF system was successfully operated for 21 cycles, demonstrating stable and robust patterns for sugar consumption and ethanol production, significantly improved productivities, and improved flocculation of yeast cells that could potentially facilitate downstream processing.

## Results

### Identification of a monitoring parameter for SCF

**Fig. 1:**
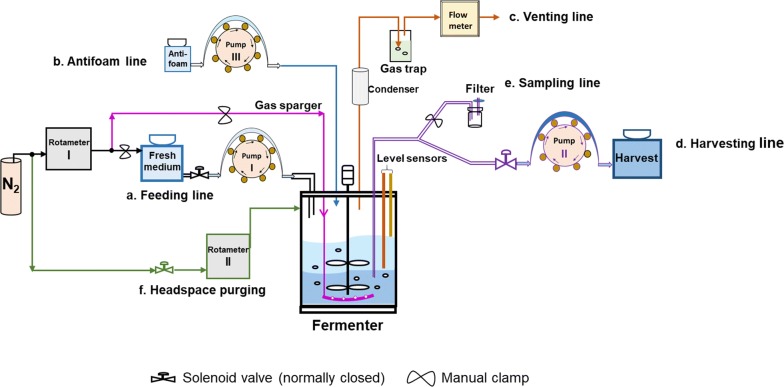
Fermenter setup for self-cycling fermentation (SCF). The temperature control and the pH probe are not shown. The control gauge for N_2_ pressure from the gas cylinder was set to 4 psig during fermentation. The various components displayed in this schematic are not necessarily represented in their actual locations

**Fig. 2:**
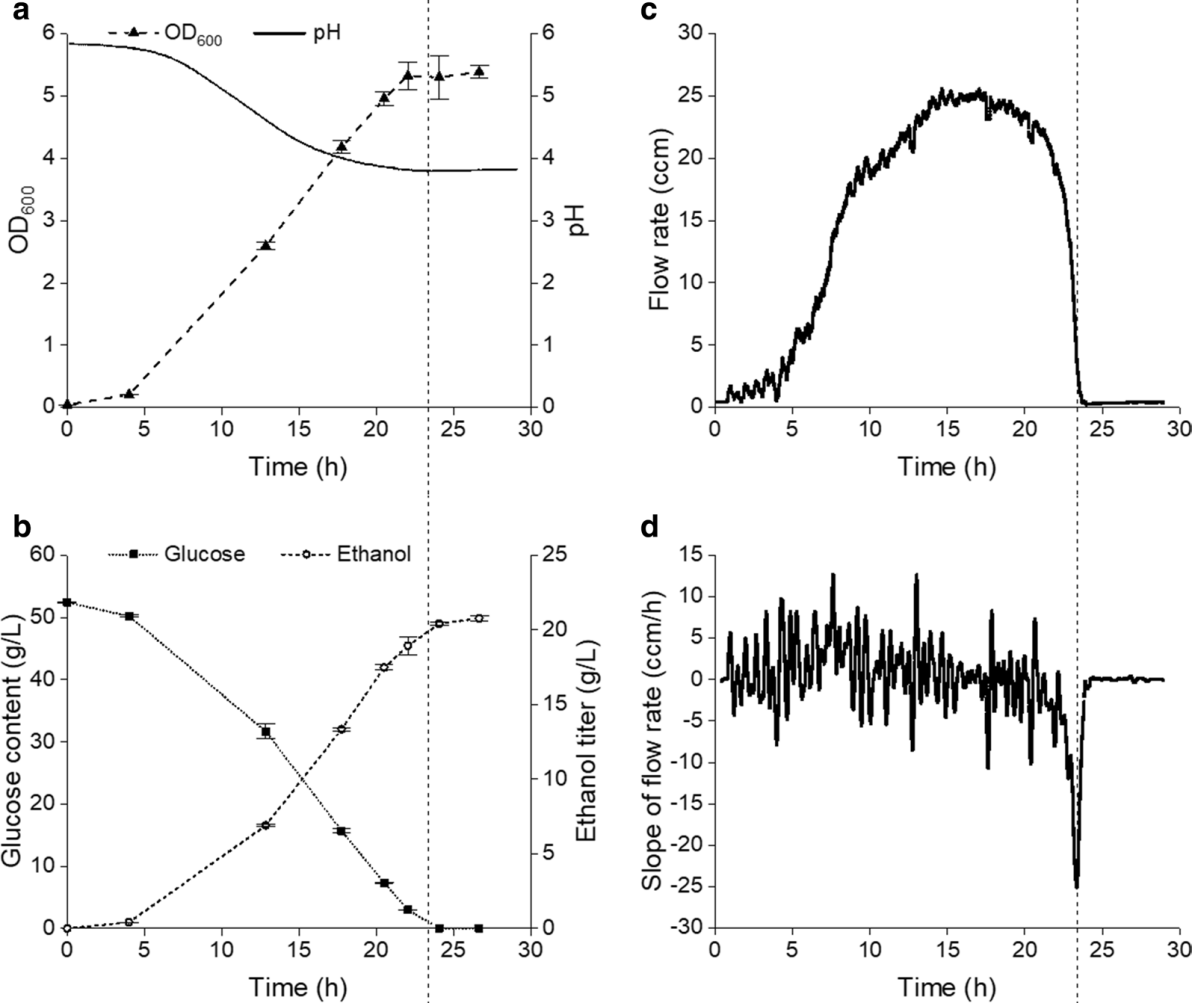
Batch fermentation. Samples were taken at different intervals during fermentation to analyze biomass contents (**a**), as well as glucose and ethanol concentrations (**b**). The data reported are the average from analytical triplicates, with error bars representing standard deviations. The pH (**a**) and flow rate of the gas released during fermentation (**c**) were monitored in real-time, and the slope of the flow rate (**d**) was automatically calculated. A vertical line was added to all graphs at 23.6 h, corresponding to the sharp minimum in slope of gas flow rate (**d**). The gas flow rate was reported in cubic centimeter per minute (ccm) at 25 °C and 1 atm, and the slope of the gas flow rate was measured in cubic centimeter per minute per cycle time (ccm/h)

Anaerobic batch fermentation was carried out using a specialized fermenter system (Fig. 1) to identify a monitoring parameter indicative of the onset of stationary phase to trigger cycling for ensuing SCF operation. As implied by Fig. 2a, typical growth patterns, characterized by lag, exponential and stationary phases, were observed for batch operation, and pH decreased below 4.0 before plateauing. During exponential growth (~ 4 to 22 h), concentrations of sugar and ethanol displayed a rapid and relatively linear change, while little or no changes were observed during the lag (~ 0 to 4 h) and stationary (~ 24 to 27 h) phases (Fig. 2a, b). Overall, considering the time at which glucose (sole carbon source) was depleted and the maxima in OD_600_ (optical density at 600 nm) and ethanol titer were reached, we can conclude that the stationary phase was reached between 22 and 24 h.

To determine whether gas released from the fermenter—essentially CO_2_ generated from the active conversion of glucose to ethanol—could be used to identify the onset of stationary phase under anaerobic conditions, a flow meter was installed at the only outlet of the fermenter (Fig. 1).

The gas flow rate and its first derivative (slope) were reported (Fig. 2c, d, respectively). It should be noted that each sampling point seen in Fig. 2a, b corresponds to a small downward spike in gas flow rate (Fig. 2c) and slope (Fig. 2d), which was caused by a small reduction in overhead gas pressure during sampling. Despite this, the flow rate of the gas venting out of the fermenter during fermentation increased to a maximum at 14–18 h and then quickly decreased (Fig. 2c). The slope of the gas flow rate (Fig. 2d) shows a sharp valley at 23.6 h (highlighted by the dotted vertical line), with a minimum lower than − 20 ccm/h. Based on the timing of this minimum and the magnitude of the slope, we identified this parameter as a potential marker of the onset of stationary phase. Therefore, the conditions for cycling in ensuing SCF operation were considered met when (1) the slope of gas flow rate fell below − 20 ccm/h and then the value continuously increased for more than 2 min; (2) the cycle time was greater than 3 h; and (3) the pH dropped below 4.0. The value of − 20 ccm/h for slope value (criterion 1) was not the minimum value observed during fermentation, but served as a transitional response to identify the onset of stationary phase. The inclusion of cycle time and pH was merely to reduce the influence of fluctuations in the slope signal (Fig. 2d).

The cumulative volume of gas evolved throughout fermentation (Additional file 1: Figure S1a) was calculated by adding the gas flow rate at a frequency of 1 s—this was similar to, yet more accurate than, the integrated area of the gas flow rate over time (Fig. 2c). Ideally, the cumulative volume of gas evolved would be proportional to the amount of sugar consumed and ethanol produced. To confirm if this was the case, we calculated the expected sugar content and ethanol titer in the fermenter based on the cumulative gas evolved for various time points, as shown in Eqs. (1) and (2), respectively.

1 $$\left[ G \right]_{t} = \left[ G \right]_{0} - \left( {\frac{{V_{{{\text{g}},t}} }}{{V_{{{\text{g}},{\text{Total}}}} }} \times \left[ G \right]_{0} } \right),$$

where [*G*]_*t*_ is the expected concentration of glucose at time *t* (g/L), [*G*]_0_ is the initial concentration of glucose (g/L), *V*_g,Total_ is the total volume of gas evolved (L), and *V*_g,*t*_ is the volume of gas evolved from the beginning of fermentation to time *t* (L).

2 $$\left[ {\text{EtOH}} \right]_{t} = \frac{{V_{{{\text{g}},t}} }}{{V_{{{\text{g}},{\text{Total}}}} }} \times \left[ {\text{EtOH}} \right]_{\text{Total}} ,$$

where [EtOH]_*t*_ is the expected concentration of ethanol at time *t* (g/L), and [EtOH]_Total_ is the final concentration of ethanol produced during the fermentation (g/L).

As shown in Additional file 1: Figure S1b and c, the predicted values obtained from Eqs. (1) and (2) showed strong correlations with the actual values determined using high performance liquid chromatography (HPLC) for glucose and gas chromatography (GC) for ethanol.

### Assessing the requirements for ergosterol and Tween 80 in anaerobic SCF

**Fig. 3:**
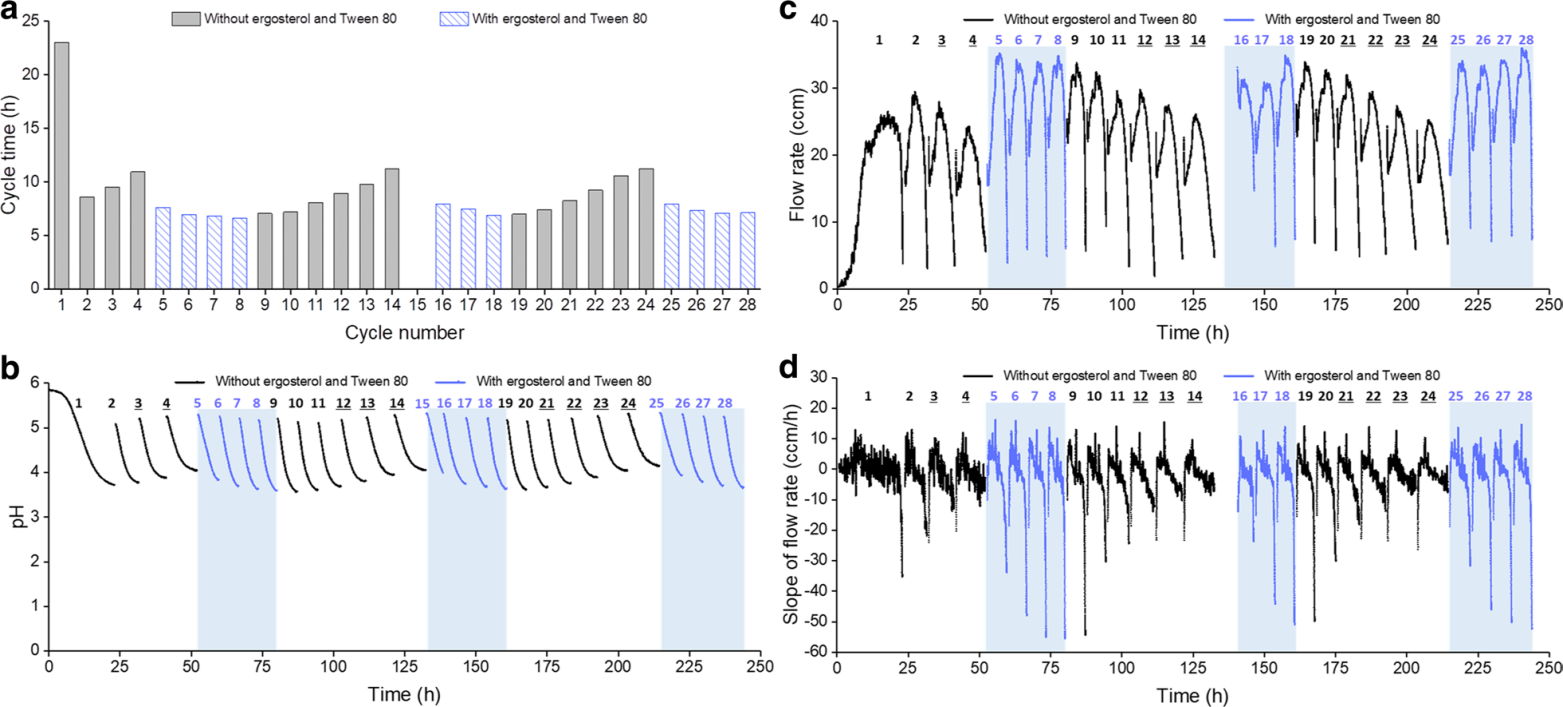
Ergosterol and Tween 80 supplementation. SCF was performed using medium without (cycles 1–4, 9–14, and 19–24) or with (cycles 5–8, 15–18, and 25–28) the supplementation of ergosterol (0.02 g/L) and Tween 80 (0.8 g/L). The cycle time (**a**), pH of the culture (**b**), as well as gas flow rate (**c**) and its slope (**d**) were reported. The cycle numbers were labeled at the top of each figure, with the exception of **a** where the cycle numbers were indicated on the *x*-axis. Cycles with supplementation were shaded in **b**–**d**. Underlined cycle numbers indicate cycles that did not meet criteria for automated cycling and that were thus manually triggered. Cycle 15 and part of cycle 16 were removed from **c** and **d** due to excessive flow of nitrogen entering the fermenter as a result of nitrogen regulator failure

The cycling conditions established in the batch experiments were used to operate SCF under the same fermenter system (Fig. 1). It has been reported in batch fermentations that sterols and unsaturated fatty acids need to be added to long-term *S. cerevisiae* cultures growing under anaerobic conditions [20, 21], as these important components of plasma membrane cannot be synthesized by yeast in the absence of oxygen [22]. In order to assess if this would apply to SCF operation, yeasts were grown in yeast nitrogen base medium (which does not contain sterols or fatty acids) for the first 4 SCF cycles, followed by 4 more cycles (cycles 5–8) in which the same medium was supplemented with ergosterol and Tween 80 (a source of unsaturated fatty acids). These were repeated for cycles 9–14 (no supplementation) and 15–18 (supplementation), as well as cycles 19–24 (no supplementation) and 25–28 (supplementation). As expected, cycle 1 behaved as a batch fermentation (Fig. 2) in terms of cycle time, pH, gas flow rate, and slope of gas flow rate (Fig. 3). However, cycle time progressively increased from cycles 2 to 4 (Fig. 3a). Supplementation with ergosterol and Tween 80 in the following cycles (5–8) resulted in a significant reduction and stabilization of cycle time. These observations were consistent in the following cycles, where exclusion of ergosterol and Tween 80 (cycles 9–14 and 19–24) progressively led to longer cycle times (Fig. 3a), higher final pH values (Fig. 3b), lower gas flow rates (Fig. 3c), and higher final slope values (Fig. 3d). Conversely, when ergosterol and Tween 80 were reintroduced to the system (cycles 15 to 18, and 25 to 28), shorter cycle times, lower final pH values, higher gas flow rates and lower minimum slope values were reinstated (Fig. 3). When multiple cycles were operated without ergosterol and Tween 80, once the cycle time increase beyond 3 h and the pH plateaued, the slope of the gas flow rate staggered and the minima of slope did not reach the setpoint to trigger cycling (see cycles 3–4, 12–14, and 21–24 in Fig. 3b, d); in those cases, cycling was manually initiated. Some residual glucose remained at the end of these cycles (between 0.1 and 0.4 g/L), whereas residual glucose was below 0.1 g/L for all other cycles (excluding 15, for which no sample was taken). Thus, cycles 3–4, 12–14, and 21–24 were manually ended earlier than the theoretical completion time, indicating that they would have a longer cycle time than reported (Fig. 3a). Interestingly, despite these small fluctuations, all cycles ended with similar final ethanol titers, other than cycles 12 and 14 where the values were lower (Additional file 2: Figure S2). In addition, it should be noted that cycle 15 and the early stages of cycle 16 are missing in Fig. 3a, c and d due to the failure of a pressure regulator in the nitrogen cylinder, which facilitated excessive flow of nitrogen into the fermenter (Fig. 1) and disrupted the readings of the gas flow meter. The flow rates and slopes for these two cycles are provided in Additional file 3: Figure S3a and b, respectively.

### Demonstration of automated SCF operation

**Fig. 4:**
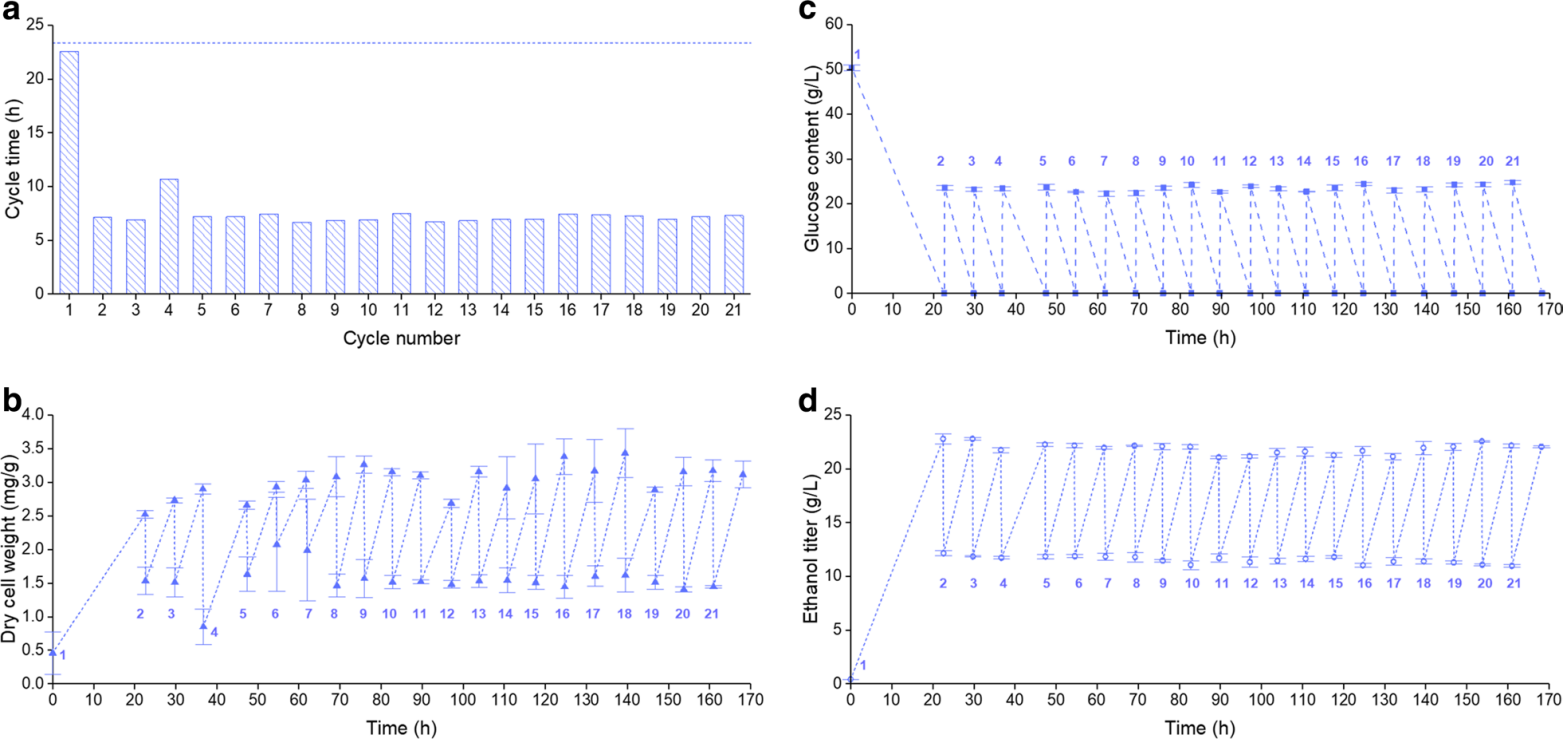
Analysis of samples from SCF demonstration. Cycle time (**a**) amount of biomass (**b**), and concentrations of glucose (**c**) and ethanol (**d**) were plotted for the beginning and end of all cycles. Medium supplemented with ergosterol (0.02 g/L) and Tween 80 (0.8 g/L) was used for cycles 1–17; the concentrations of ergosterol and Tween 80 were tripled for the remaining cycles. Cycle 4 was disrupted by halting agitation and temperature control for ~ 7 h. Cycle numbers were labeled at the initial stage of each cycle, with the exception of **a** where the cycle number was indicated on the *x*-axis. The horizontal line in **a** represents the fermentation time for batch operated under similar conditions (Additional file 4: Table S1). In **b**–**d** data are reported as the average from at least three analytical replicates, with error bars representing standard deviations

SCF was operated for 21 consecutive cycles with the supplementation of ergosterol and Tween 80 to test whether the system is stable, robust, and able to improve productivity (Fig. 4). The agitation and temperature control were interrupted during cycle 4 to evaluate the capacity of the system to recover from disturbances, and the concentrations of ergosterol and Tween 80 were tripled in cycles 18–21 to determine if excess would impact fermentation. A batch experiment with the supplementation of ergosterol and Tween 80 was performed under the same conditions as SCF for comparison (Additional file 4: Table S1 and Additional file 5: Figure S4).

Figure 4 shows the main parameters analyzed over SCF operation. First, the cycle time (Fig. 4a) for cycles 2–21 was consistent for all cycles (except cycle 4, for which agitation and temperature control were halted). It ranged from 6.7 to 7.5 h, which was approximately 1/3 the duration of cycle 1 and batch experiment (Additional file 4: Table S1). Biomass production, as measured by dry cell weight, was also relatively consistent for all cycles—except cycles 4 and 11—with a starting concentration of ~ 1.5 mg/g culture and reaching ~ 3 mg/g at the end of cycles (Fig. 4b). As seen in Fig. 4c, d, the changes in concentrations of glucose and ethanol generated regular patterns. Starting from cycle 2, glucose went from 23.4 to 0 g/L over the duration of a cycle, while ethanol went from 11.5 to 21.9 g/L. Fermentation efficiency was calculated by comparing the ratio of glucose consumption (g/L) through a cycle over its ethanol production (g/L) against a theoretical value of 0.51. As shown in Additional file 4: Table S1, fermentation efficiency fluctuated among SCF cycles, ranging from 80.8 to 91.2%, but were similar or greater than the efficiency achieved in batch operation (81.1 ± 0.8%). Note that ~ 2.2 g ethanol was detected in the gas trap at the end of the whole fermentation, implying that some ethanol evaporated from the culture over the 21 cycles (168.1 h). Thus, the total amount of ethanol produced and the fermentation efficiency could be greater than values found in Fig. 4d and Additional file 4: Table S1, respectively. In addition, glycerol is commonly produced during ethanol fermentations as a means to balance redox potential in the cell, particularly when yeast are grown in stressful conditions [23]. Our analytical results showed that glycerol was present at the beginning of cycles 2–21 at a concentration of 1.8–2.0 g/L and accumulated to 3.3–3.5 g/L over a cycle. In comparison, while no glycerol was detected at the onset of cycle 1 and batch experiment performed with the same medium, it accumulated to 3.6 g/L. Finally, high concentrations of organic acids are typically attributed to bacterial contamination [23]. Lactic acid was not detected throughout SCF operation; acetic acid concentration was less than 0.8 g/L; and no contamination was observed under microscope. Thus, contamination was likely not an issue during the long-term fermentation campaigns performed in this study.

**Fig. 5:**
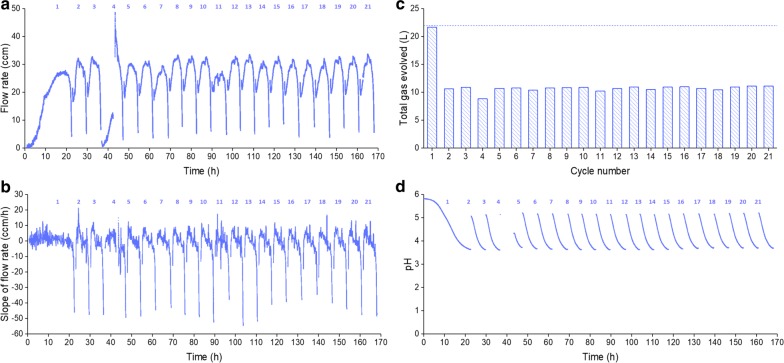
Online monitoring parameters from SCF demonstration. Gas flow rate (**a**), slope of gas flow rate (**b**), total volume of gas evolved per cycle (**c**), and pH (**d**) were monitored throughout SCF operation. Medium supplemented with ergosterol (0.02 g/L) and Tween 80 (0.8 g/L) was used for cycles 1–17; the concentrations of ergosterol and Tween 80 were tripled for the remaining cycles. Cycle 4 was disrupted by halting agitation and temperature control for ~ 7 h. Cycle numbers were labeled at the top of each figure, with the exception of **c** where the cycle numbers were indicated on the *x*-axis. The horizontal line in **c** represents the total volume of gas evolved in batch operation conducted under similar conditions (Additional file 5: Figure S4c)

Figure 5 shows the online monitoring data related to gas flow rate and pH during SCF operation. Starting from cycle 2, gas flow rate curves (Fig. 5a), except for cycles 4 and 11, were generally sharper and narrower than that of cycle 1, suggesting a faster production rate of CO_2_. It should be noted that the gas flow rate curves for cycles 4 and 11 were considered outliers because of an intentional disruption to the system (see below) and intracycle sampling that relieved system pressure, respectively. For all 21 cycles, after cycle time passed 3 h and pH dropped below 4 (Fig. 5d), slope values fell below − 20 ccm/h and then increased for over 2 min (Fig. 5b). Therefore, cycling criteria were met, and all the cycles were automatically driven throughout the SCF operation. Total volumes of gas evolved for cycles 2–21 were approximately half the value for cycle 1 and batch operation (Fig. 5c). The scale of pH change (Fig. 5d) observed over a cycle was repeatable for cycles 2–21 throughout the entire fermentation. The reproducibility of these patterns highlights the stability of the system.

As mentioned above, agitation and temperature control were interrupted for ~ 7 h in cycle 4 to test the capacity of the SCF system to recover from disturbances. This resulted in an abnormal trend of gas flow rate (Fig. 5a), lower total volume of gas evolved (Fig. 5c), and a longer cycle time (Fig. 4a), but the pH (Fig. 5d) and the slope of the gas flow rate (Fig. 5b) eventually reached similar levels as other cycles. More importantly, the cycling process was still automatically triggered to initiate cycle 5, which behaved similarly to cycles 2 and 3. This implies that the system is robust enough to withstand interruptions or variations in operating conditions.

Since the addition of ergosterol and Tween 80 was based on concentrations referred by Straver et al. [24], starting from cycle 18, the contents of ergosterol and Tween 80 were increased threefold to see whether this would have a positive or negative impact on fermentation. No substantial difference in fermentation parameters could be observed (Figs. 4 and 5), indicating that the initial concentrations of ergosterol and Tween 80 were already at or above the optimal levels for cell growth. In addition, as cycle number increased, cells progressively aggregated to the surfaces of probes and fermenter wall above culture and flocculation became obvious (Additional file 7: Figure S6), which was not observed during batch or cycles 1–3 of SCF. This also applied to the previous experiments accessing the requirement of ergosterol and Tween 80 (results shown on Fig. 3).

To explore the changes occurring during an SCF cycle, intracycle samples were taken throughout cycles 2 and 11. Although more samples at an increased frequency would be needed to better define the details of the kinetics, Additional file 6: Figure S5b and d revealed that the consumption of glucose and production of ethanol were generally linear in both cycles. This would suggest that cells at the end of each SCF cycle were able to quickly uptake nutrients and produce ethanol as the limiting nutrient became available again. However, dry cell weight (Additional file 6: Figure S5a and b) did not readily increase at the early stages of the cycles, which might be contributed by the experimental errors related to the measurement and cell flocculation. Future work is needed to investigate the reason for this.

### Improvements in productivity

**Fig. 6:**
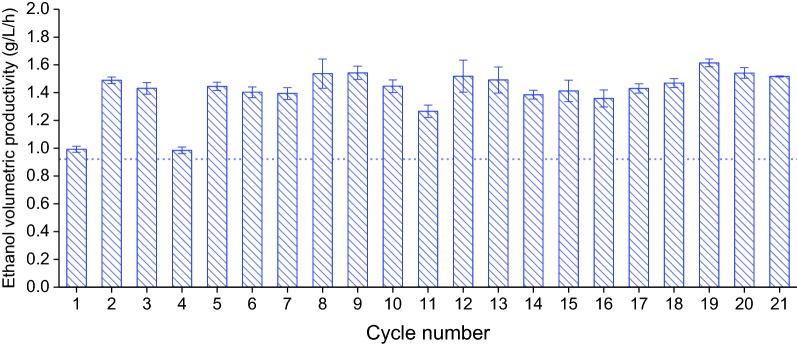
Ethanol volumetric productivity during SCF. Medium supplemented with ergosterol (0.02 g/L) and Tween 80 (0.8 g/L) was used for cycles 1–17; the concentrations of ergosterol and Tween 80 were tripled for the remaining cycles. Cycle 4 was disrupted by halting agitation and temperature control for ~ 7 h. The horizontal line represents the volumetric productivity in batch operation conducted under similar conditions. The data reported are the average from at least three analytical replicates, with error bars representing standard deviations

Ethanol volumetric productivity represents the ethanol produced in a cycle per unit volume per cycle time. As seen in Fig. 6, SCF operation led to improvements of 37.5–75.3% compared to batch, excluding cycles 1 (7%), which was essentially a batch, and 4 (8%), for which agitation and temperature control were interrupted. Annual ethanol productivity (Additional file 4: Table S1) refers to the total amount of ethanol that can be potentially produced over a year, in this case, for a fermenter assumed to be at 10^5^ L scale and including conditions such as harvesting time and cleaning time [5]. As seen in Additional file 4: Table S1, switching production from batch to 21-cycle SCF campaigns, annual ethanol productivity could be increased by 75.8 ± 2.9%.

## Discussion

### The monitoring parameter for anaerobic SCF

In batch fermentation, the production of ethanol, a primary metabolite, showed little change at lag phase and plateaued at stationary phase (Fig. 2a, b). Hence, the application of SCF operation, in which cycling is triggered at the onset of stationary phase, is expected to minimize the time for which ethanol is not produced. In order to trigger cycling process upon the arrival of stationary phase, it is important to define an appropriate monitoring parameter capable of doing so [11].

Common vibrational spectroscopes for online monitoring of ethanol fermentation have limitations for application in SCF. For instance, for near-infrared and Raman spectroscopic techniques, the fermentation culture needs to be filtered or precipitated to reduce interference from solids present in suspension [16, 18], an approach which is not practical in industry. The use of Fourier transform infrared spectroscopy by Veale et al. [17] required the installation of an Attenuated Total Reflectance flow cell to the reactor, making part of the fermentation culture continuously circulate between the fermenter and the flow cell. This increases the risk of contaminating cultures and of solids/cells blocking the circulation path. CER has been used as a monitoring parameter for SCFs performed under aerobic conditions [9, 10]. Although well studied, CER was mainly determined using infrared radiation sensors assessing the concentration of gaseous CO_2_ and dilution of the gas outlet streams was necessary to avoid saturation of the signal. In our anaerobic system, CO_2_ was the only gas evolved, which would also saturate the sensor, and these sensors were thus not used in this study.

As an alternative, we used a gas flow meter (Fig. 1) to record the flow rate of the gas evolved and calculate the slope of flow rate in real time. As can be seen, the slope of the gas flow rate was an appropriate monitoring parameter to identify the onset of stationary phase (Fig. 2). We demonstrated how these conditions could be used to efficiently automate SCF operation under anaerobic conditions (Fig. 5). Furthermore, a gas flow meter does not enter in contact with liquid cultures, eliminating probe fouling issues [9] and reducing risks of contamination raised by possible repair/replacement of the meter during fermentation. It is also relatively cheap for industrial purposes and only needs to be connected to the venting line, without significant changes to existing infrastructure.

It should be noted that the flow rate of gas leaving the reactor recorded by the flow meter may not be equal to the real production rate of CO_2_ by yeast. This is primarily because gas CO_2_ was mixed with N_2_ at the beginning of each SCF cycles to ensure anaerobic conditions. In this case, ~ 3 L of N_2_ left the fermenter in the early stages of the cycles, making the early flow rate readings (Figs. 2c, 3c and 5a) approximately 84% of the actual values, since the flow meter was calibrated with pure CO_2_ while the gas contained N_2_. This influenced the calculation of flow rate by Poiseuille equation. As the amount of CO_2_ produced by the yeast increased during the fermentation (e.g., a total volume of approximately 22 L CO_2_ at the end of batch; Additional file 1: Figure S1a and Additional file 5: Figure S4c), N_2_ was driven out of the headspace, eventually nearing zero.

Initially designed to trigger cycling, the monitoring parameter—evolved gas flow rate—was found to have the capability of providing a quick estimation of sugar and ethanol concentrations inside the fermenter, as well as information on different fermentation scenarios. We observed that the concentrations of sugar and ethanol in batch fermentation (Fig. 2b) correlated well with values predicted from the cumulative gas volume (Additional file 1: Figure S1b and c), in spite of N_2_ being added to the system at the beginning of the operation. While more data should be gathered to further verify the strength of the correlation, this could provide very useful real-time information on the status of ethanol fermentations. Practically for industry, it shows great promise in helping reduce labor intensity in sampling preparation and analysis, as well as the use of expensive equipment, such as HPLC/GC for fermentation monitoring [15]. In addition to that, SCF cycles 4 and 11 during the demonstration study had lower flow rate peaks than the other cycles (Fig. 5a). The former saw slower fermentation due to a lower temperature and no agitation, and the latter was disturbed by frequent sampling. Furthermore, SCF cycles without supplementation of ergosterol and Tween 80 (cycles 3–4, 12–14, and 21–24 in Fig. 3) had less pronounced values in final slopes of flow rate (Fig. 3d), aligning with slower growth in the absence of ergosterol and fatty acids. Finally, it is worth noting that although one of the cycling criteria employed successfully in these experiments was the decrease of the flow rate slope below − 20 ccm/h, fermentation systems using different organisms or sugars (i.e., sugar types and concentrations) may require alternative values for robust cycling.

### Assessing the requirements for ergosterol and Tween 80 in anaerobic SCF

The use of non-supplemented medium in anaerobic SCF of *S. saccharomyces* led to a progressive reduction in growth rate, as seen in the extension of cycle times over multiple cycles (Fig. 3a). This was not observed in a previous study in which five successive cycles were performed in shake flasks fitted with S-locks for ethanol fermentation [5]. We posit that, because the cycling was performed manually with shake flasks, which briefly exposed the culture to air [5], small amounts of O_2_ present at the beginning of each cycle were sufficient for the yeast to synthesize the sterols and unsaturated fatty acids necessary for constructing plasma membrane [25]. In our current study using a 5-L fermenter, the batch experiment and cycle 1 of SCF operation were inoculated with yeast cells that had been cultivated aerobically, and sterols and fatty acids were re-distributed into daughter cells upon division [22]. By the end of batch fermentation or cycle 1 in SCF, cells had divided approximately 6 times (data from cell counts with hemocytometer), which exceeds the 4–5 generation limit for daughter cells to still benefit from parental sterol and fatty acids under anaerobic conditions [22]. Consequently, the yeast cells for subsequent cycles had diminishing levels of sterols and unsaturated fatty acids, putting them under increasing stress and leading to longer cycle times (cycles 2–4 in Fig. 3a). By supplying ergosterol and Tween 80 (a source of unsaturated fatty acids), the growth rate quickly increased, even for cycle 5, likely due to recovering integrity of the plasma membrane and improving transportation of chemicals. The fact that the absence and supplementation of ergosterol and Tween 80 were directly related to slower and faster growth, respectively, demonstrates the importance of these compounds for sustained SCF operation under anaerobic conditions. It should also be noted that the addition of ergosterol and Tween 80 to batch fermentation did not impact ethanol production (Additional file 2: Figure S2). Finally, the ethanol titers observed for cycles 12 and 14 were lower than for other cycles (Additional file 2: Figure S2). The precise explanation for this is not known. Nevertheless, the ethanol titers reached in these cycles were still high (above 17 g/L). Interestingly, lower ethanol titers were never observed in cycles where ergosterol and Tween 80 were supplemented.

From an industrial perspective, it would be interesting to conduct studies investigating the addition of small amount of air at the beginning stage of each cycle to initiate the synthesis of sterol and unsaturated fatty acids. This would be a much cheaper option than the addition of ergosterol and Tween 80.

### Demonstration of automated SCF operation

Our previous proof-of-concept study suggested that operating SCF campaigns for ~ 20 cycles would significantly improve annual ethanol productivity, while maintaining a low risk of contamination [5]. Therefore, we performed automated SCF for 21 cycles and tested whether productivity improved accordingly compared to batch.

The first thing to note is that, in comparison to batch fermentation and cycle 1, both the ethanol volumetric productivity (Fig. 6) and the annual ethanol productivity (Additional file 4: Table S1) are significantly greater under SCF operation. The increase in annual ethanol productivity from SCF operation (75.8 ± 2.9%, Additional file 4: Table S1) was similar to the value predicted in the lower scale proof-of-concept study (62.7 ± 11.9%) [5]. It should be noted that annual ethanol productivities were calculated based on operation of the 5-L fermenter. Thus, the value of the reported annual ethanol productivity (Additional file 4: Table S1) may differ for real industrial conditions as many parameters would change upon scale-up. Nevertheless, the significant improvement of SCF over batch operation with regards to annual ethanol productivity highlights the potential of SCF for ethanol production. Furthermore, it is important to point out that both fermentation efficiency (Additional file 4: Table S1) and improvement in productivities confirm the great potential of automated SCF operation at larger scale.

Ethanol volumetric productivity remained relatively stable among cycles 2–21, except for cycle 4 (Fig. 6). Small discrepancies were likely due to typical processing fluctuations in operating conditions (temperature, concentrations of nutrients, and feeding and harvesting times). Cycle 11 in particular shows variations that were likely due to disturbances from a significantly larger number of samples taken to perform intracycle analysis (Figs. 4, 5, 6, and Additional file 6: Figure S5). This disturbed cultures and readouts and decreased the working volume. Therefore, compared to cycles 2–21 (except 4), a lower value in maximum flow rate (Fig. 5a), slightly longer cycle time (Fig. 4a), and lower ethanol volumetric productivity (Fig. 6) were found for cycle 11. Despite this, SCF operation was stable and robust, and production was reproducible and efficient.

It should be noted that our study used a sugar concentration of 50 g/L, which is lower than the concentrations used in many bioethanol plants. This study thus serves as a stepping stone, providing the first demonstration and establishing the basis for the use of SCF for ethanol production. We expect this study will open the door to future work investigating more parameters and variables affecting SCF operation and ethanol production, such as the use of higher levels of sugars (e.g., 100 g/L) and of real cellulosic hydrolysates.

Unlike in batch and early SCF cycles, cell aggregation—deposition to solid surfaces and flocculation—was observed in later SCF cycles (Additional file 7: Figure S6). This has not been reported in prior studies of any SCF systems using yeast. Although the exact cause of the cell aggregation, which lasted until the end of operation, is not clearly established, Ma et al. [26] reported that a repeated-batch method (in which 60% of the culture was drained and refilled every batch) was used to improve flocculation of *S. cerevisiae*. In that study, approximately 18 sequential batches were necessary for yeast to reach high levels of flocculation, compared to the first 10 batches which saw much less flocculation [26]; whereas in our study apparent flocculation was observed as early as cycle 4. It is also interesting that the ethanol volumetric productivity (Fig. 6) was not affected by flocculation, aligning with results of increasing productivity from Ma et al. [26]. According to Guo et al., yeast genes associated with flocculation, such as FLO 1, 5, 9, and 10, are primarily responsible for cell–cell aggregation [27], and it is possible that our SCF process could have activated such genes. As Soares [28] points out, the optimal pH for yeast flocculation is generally between pH 3 and 5, depending on the strain, hence pH may have also played a role in the aggregation observed (Fig. 5d). Since cell aggregation in SCF rendered biomass quantification by common approaches difficult and unreliable, dry cell weight, rather than OD_600_ and cell counting, was used. Because of this, it was not possible to clearly assess the level of cell synchrony achieved, which is usually determined by cell counts [9].

Finally, flocculation may have many industrial advantages such as facilitating downstream processing. The quick settling of cells at the end of later SCF cycles improved separation and could lead to great reduction in energy requirements for filtration, which is commonly used before distillation [29]. The recovered cells could be re-used for subsequent fermentation campaigns, enabling cell aggregation in all future SCF cycles, again facilitating downstream processing.

## Conclusions

To our knowledge, this is the first report of successful and sustainable operation of automated SCF under anaerobic conditions, as well as the first SCF operation driven by gas flow measurements. Clearly, this study demonstrates that stable and robust operation of anaerobic SCF is achievable, and leads to improved productivities, primarily due to reduced fermentation time and down time, while maintaining a similar ethanol titer throughout operation. Together with flocculation, this can potentially contribute to significant reductions in capital and operational costs for both fermentation and downstream processes. Overall, with its defined medium and operating conditions, the SCF demonstration in this work can be taken as a starting point from which future investigations and industrial applications can be built.

## Methods

### Media and yeast

Two types of medium were used for fermentations. Yeast nitrogen base (YNB) medium [50 g/L glucose and 6.7 g/L yeast nitrogen base with amino acids in 0.1 M sodium phosphate buffer (pH 6.0)] was filter-sterilized into a 10-L carboy (Nalgene™, Thermo Fisher Scientific, Waltham, MA, USA). The second medium consisted of YNB medium supplemented with 0.02 g/L ergosterol (Sigma-Aldrich, St. Louis, MO, USA) and 0.8 g/L Tween 80 (Sigma-Aldrich, St. Louis, MO, USA) [24]. The protocol for adding ergosterol and Tween 80 was adapted from Andreasen and Stier [20]. Briefly, ethanol was mixed with ergosterol and Tween 80, and the mixture was boiled until the solution was clear. This was then mixed with YNB medium (filter-sterilized) for a homogenous emulsion. Note that the resulting concentration of ethanol in the second medium was below 0.5 g/L.

The industrial yeast strain *Saccharomyces cerevisiae* Superstart™, purchased from Lallemand Ethanol Technology (Milwaukee, WI, USA), was used for this study. Yeast cells were cultivated and aliquoted into multiple glycerol stock vials and stored at − 80 °C until use. Prior to each fermentation run, a new vial was thawed, streaked on solid agar (14 g/L, Thermo Fisher Scientific, Waltham, MA, USA) plates containing yeast extract peptone dextrose (50 g/L, Thermo Fisher Scientific, Waltham, MA, USA), incubated at 30 °C for 2 days, and then stored at 4 °C for 1–2 days prior to fermentation. Seed cultures were prepared according to [5], and inoculation was made by transferring seed cultures from 500-mL shake flask into the fermenter at 8% (v/v) of the fermentation medium.

### Fermentation system

A 5-L fermenter (Infors-HT, Bottmingen, Switzerland) equipped with a heating jacket, condenser, high and low conductivity level sensors, antifoam pump (III), temperature control, gas sparger, rotameter II and impellers was used for all fermentation experiments in this study (Fig. 1). An EasyFerm pH probe (Hamilton Company, Reno, NV, USA) was also attached to the fermenter. All gas inlets and outlets were coupled with 0.2-µm filters (PTFE; Sigma-Aldrich, St. Louis, MO, USA). The fermenter (Fig. 1) was connected with hardware for feeding (a), harvesting (d), sampling (e), and gas purging to headspace (f). Two stainless steel rods, placed at the 1-L and 2-L levels (with error < 1.5%) when the mixing rate was kept at 200 rpm, were used as conductivity level sensors in the fermenter. The whole system, as shown in Fig. 1, was controlled by a scheme developed in the Labview^®^ environment (National Instrument, Austin, Texas, USA).

#### Feeding line (a)

A 10-L carboy containing fresh sterile medium was constantly mixed to supply fresh medium to the fermenter. N_2_ (99.998% purity; Praxair Canada Inc, Mississauga, ON, Canada) was used to purge the carboy prior to and during feeding of fresh medium to the fermenter to minimize the possibility of O_2_ entering the system. Fresh medium was transferred at a flow rate of 300 mL/min using a peristaltic pump (I; MasterFlex^®^ L/S^®^ drive, Head model: 77200-60; Cole-Parmer, Montreal, QC, Canada).

#### Antifoam line (b)

Antifoam Y-30 emulsion (Sigma-Aldrich, St. Louis, MO, USA) was sent to the fermenter through a peristaltic pump (III; Infors-HT; Bottmingen, Switzerland) to a final concentration of ~ 100 µL/L culture.

#### Venting line (c)

During fermentation, N_2_ and/or CO_2_ gas leaving the fermenter were vented through a condenser kept at 15 °C, followed by a gas flow meter (Whisper series, MW-200SCCM-D/5M; Alicat Scientific, Inc., Tucson, AZ, USA), which measured the temperature, pressure, flow rate, and integrated flow rate of the gas, before being released to the atmosphere. The flow meter was calibrated with pure CO_2_ by the manufacturer. A gas trap with distilled water was placed between the condenser and the gas flow meter to ensure that air would not flow back into the fermenter. Note that all values of gas flow rate are reported at 25 °C and 1 atm.

#### Harvesting/sampling line (d/e)

The same port, divided by a Y-shape tube (e), was used for both functions. Harvesting was performed using a digital peristaltic pump (II; MasterFlex^®^ L/S^®^ drive, Head model: 77201-60; Cole-Parmer, Montreal, QC, Canada) at a set flow rate of 300 mL/min, with the sampling line clamped. Sampling was achieved by creating a vacuum using a syringe attached to the filter (e). After sampling, samples were transferred to sterile 15-mL centrifugation tubes, and immediately stored at − 80 °C until offline analysis was performed.

#### Headspace purging (f)

During cycling of SCF (see “Cycling process” section), N_2_ was sent to the headspace of the fermenter. This balanced the pressure during harvest, minimized entry of O_2_ during feeding, and minimized losses of ethanol, the volatile product.

#### Batch fermentation

Sterile medium was added to the fermenter though the feeding line (a), heated to 30 °C, and flushed with N_2_ through the gas sparger for 30 min. After adding antifoam (b), the fermentation was initiated by inoculation and the fermenter was flushed with N_2_ again for 10 min. The fermenter was incubated at 30.0 ± 0.2 °C, with an impellor mixing rate of 600 ± 2 rpm. For all fermentations performed in this study, a 2-L working volume was used.

#### SCF operation

The fermenter was controlled at the same conditions as for batch operation, except that at the onset of stationary phase, the cycling process was automatically performed.

#### Cycling criteria

The criteria to initiate cycling were set in the Labview^®^ program as follows: cycle time > 3 h, pH < 4.0, and slope of the outlet gas flow rate reached a value lower than − 20 ccm/h, which then continuously increases for 2 min. The rational for these criteria can be found in “Results” section.

#### Cycling process (harvest and feed)

When the cycling criteria were met, the impellor mixing rate dropped to 200 rpm to ensure accurate signal from level sensors, after which half the culture was withdrawn through the harvest line (d), while N_2_ was automatically purged through line (f) to balance the pressure of the fermenter’s headspace. When the 1-L level was reached, harvesting was stopped and fresh medium was fed through the feeding line (a) to start a subsequent cycle. Once feeding was complete (2-L level was reached), agitation returned to 600 rpm, antifoam was added, and the N_2_ purge continued for another 5 min. Finally, data logging started. Despite the fact the program was designed to automatically run the cycling process, it was possible to manually initiate cycling even if the criteria were not met (for example, if the operator saw the pH plateau at ~ 4, the slope of outlet gas flow rate reached a minimum (yet not as low as − 20 ccm/h) and staggered with a continuous increase for over 2 min). Samples were taken during harvesting and after antifoam addition to represent the cultures at the end and beginning of cycles.

#### Cycle time

For batch, cycle time was defined as the time from inoculation to the point when the minimum in the slope of gas flow rate occurred. For cycle 1 of SCF, the cycle time covered from inoculation to the end of harvest; whereas for all the other SCF cycles, it was the period from start of feeding to the end of harvest.

#### Data logging

During fermentation, time, temperature, pH of the culture, flow rate of the outlet gas, slope of the gas flow rate, and integrated gas flow rate were calculated and recorded every 10 s. Flow rate in this report was exported as the average value over a 15-min time span. Slope of gas flow rate was calculated from a linear regression of the data over the same time span. Finally, integrated gas flow rate was calculated every 1 s as the cumulative flow rate.

#### Sampling

Sampling was performed as described above. For the batch fermentation, since no obvious flocculation was observed, samples were analyzed for OD_600_ using a UV–Vis spectrometer (Ultrospec 4300 Pro; Amersham Biosciences, Mississauga, ON, Canada) [5]. For samples from SCF demonstration (21 cycles), due to flocculation, dry cell weight was performed by centrifuging the cells at 10,016×*g* (accuSpin™ 400; Thermo Fisher Scientific, Waltham, MA, USA) for 10 min, replacing the supernatant—which was kept for liquid and gas chromatography analyses—with ~ 10 mL 0.01 mol/L sterile sodium phosphate buffer (pH 6.0), and centrifuging the cells a second time. The supernatant was then discarded and the pellet was re-suspended in deionized water, placed in a pre-weighed dish, left to dry in an oven at 60 °C until the mass stabilized. Dry cell weight was calculated using Eq. (3):

3 $${\text{Dry cell weight}} = \frac{{{\text{dry weight of the dish with sample}} - {\text{dry weight of empty dish }}}}{{{\text{weight of centrifuge tube with culture sample}} - {\text{weight of empty centrifuge tube}}}}.$$

The supernatants of thawed centrifuged samples were analyzed by HPLC and GC [30]. For glucose content below 1 g/L, a d-glucose kit was used [5]. Samples were also analyzed by microscopy throughout the study to check contamination.

## Supplementary information

**Additional file 1: Figure S1.** Relationship between cumulative gas flow and fermentation parameters. The cumulative gas flow (a), as well as the predicted and measured contents of glucose (b) and ethanol (c) were plotted. The dotted lines in (b) and (c) represent ideal scenario where predicted values were equal to the measured ones.

**Additional file 2: Figure S2.** Final ethanol titer at the end of each SCF cycle. SCF was performed using medium without or with the supplementation of ergosterol (0.02 g/L) and Tween 80 (0.8 g/L). The data reported is the average of analytical triplicates, with error bars representing standard deviations. No samples were collected for the end of cycle 15.

**Additional file 3: Figure S3.** Ergosterol and Tween 80 supplementation for cycle 15 and 16. Medium with supplementation of ergosterol (0.02 g/L) and Tween 80 (0.8 g/L) was used for these two cycles. Cycle numbers were labeled at the top of each figure. Cycle 15 and part of cycle 16 were removed from c and d, due to excessive flow of nitrogen to the fermenter as a result of nitrogen regulator failure; however, they are displayed here for gas flow rate (a) and slope of gas flow rate (b).

**Additional file 4: Table S1.** Comparison between batch and SCF operation under similar conditions.

**Additional file 5: Figure S4.** Batch fermentation with supplements. Medium supplemented with ergosterol (0.02 g/L) and Tween 80 (0.8 g/L) was used. Gas flow rate (a), slope of gas flow rate (b), total gas captured per cycle (c), and pH (d) were monitored throughout SCF operation.

**Additional file 6: Figure S5.** Intracycle sampling during SCF demonstration. Biomass dry cell weight, glucose and ethanol concentrations were plotted for cycle 2 (a and b) and 11 (c and d). Medium supplemented with ergosterol (0.02 g/L) and Tween 80 (0.8 g/L) was used. The data reported is the average of analytical triplicates, with error bars representing standard deviations.

**Additional file 7: Figure S6.** Cell deposition and flocculation observed during SCF. A picture was taken at the end of cycle 21 (cycle time of 6.4 h) for a demonstration of cell deposition (a). Pictures were also taken at 0 s (b) and 30 s (c) after the impellors were completely stopped for cycle 21 to show the effect of flocculation.

## Data Availability

All data generated or analyzed during this study are included in this published article and its additional files.

## Abbreviations

SCF: self-cycling fermentation
CER: carbon dioxide evolution rate
OD_600_: optical density at 600 nm
ccm: the unit of gas flow rate, cubic centimeter per minute
ccm/h: the unit for slope against cycle time, cubic centimeter per minute per cycle time
[*G*]_*t*_: the concentration of glucose at time *t* (g/L)
[*G*]_0_: the initial concentration of glucose (g/L)
*V*_g,Total_: the total volume of gas evolved (L)
*V*_g,*t*_: the volume of gas evolved from the onset of fermentation to time *t* (L)
[EtOH]_*t*_: the concentration of ethanol at time *t* (g/L)
[EtOH]_Total_: the final concentration of ethanol produced during the fermentation (g/L)
YNB: yeast nitrogen base
HPLC: high-performance liquid chromatography
GC: gas chromatography
