# Supplementary figures and images for: Improved bioethanol productivity through gas flow rate-driven self-cycling fermentation
Source: Biotechnol Biofuels. 2020 Jan 24;13:14. doi: 10.1186/s13068-020-1658-6 (PMC6979077; doi:10.1186/s13068-020-1658-6)

## Slide 1
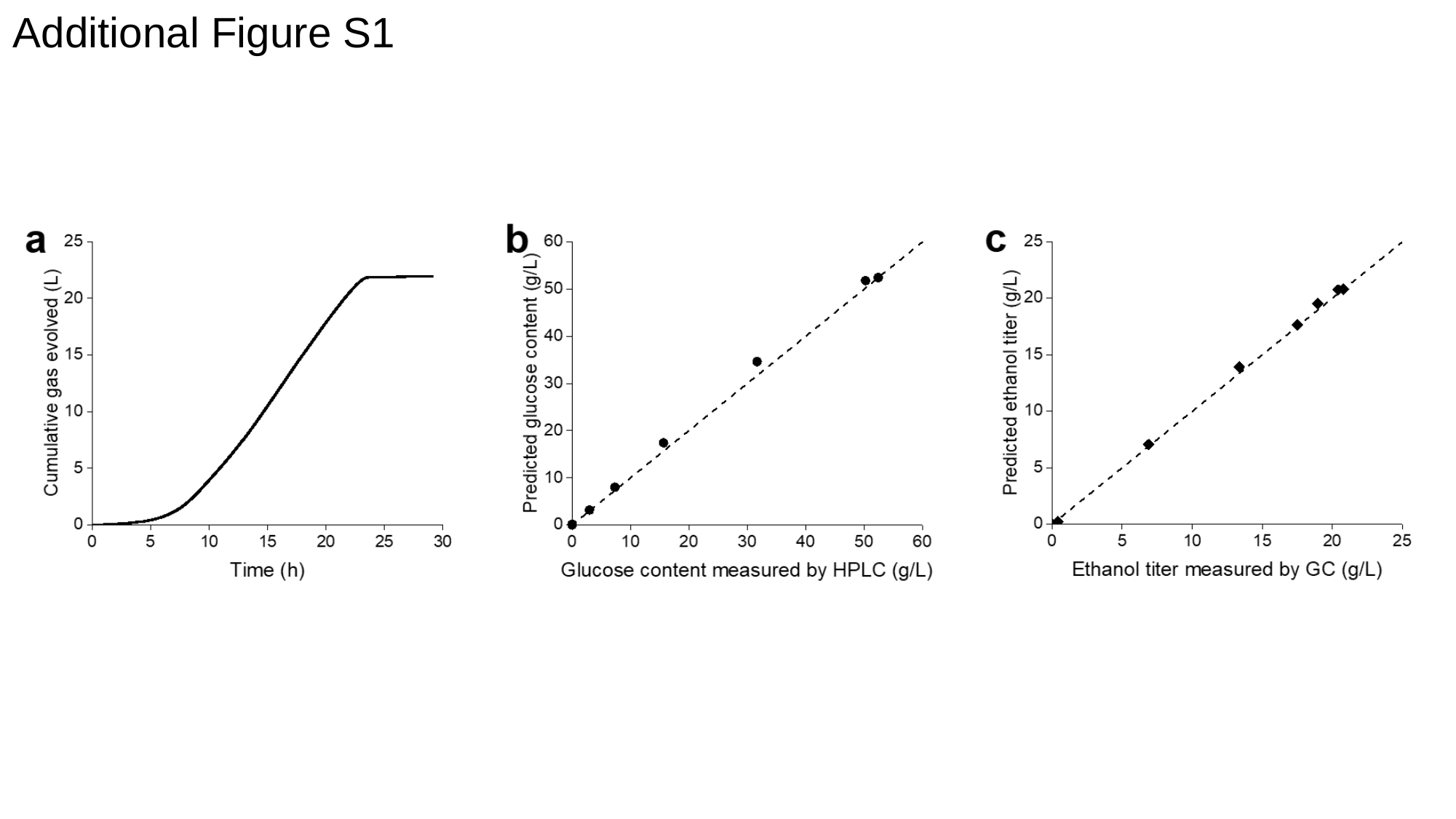

Additional Figure S1

Supplement: Supplementary file 1 — Additional file 1: Figure S1. Relationship between cumulative gas flow and fermentation parameters. The cumulative gas flow (a), as well as the predicted and measured contents of glucose (b) and ethanol (c) were plotted. The dotted lines in (b) and (c) represent ideal scenario where predicted values were equal to the measured ones. [file 13068_2020_1658_MOESM1_ESM.pptx]

## Slide 1
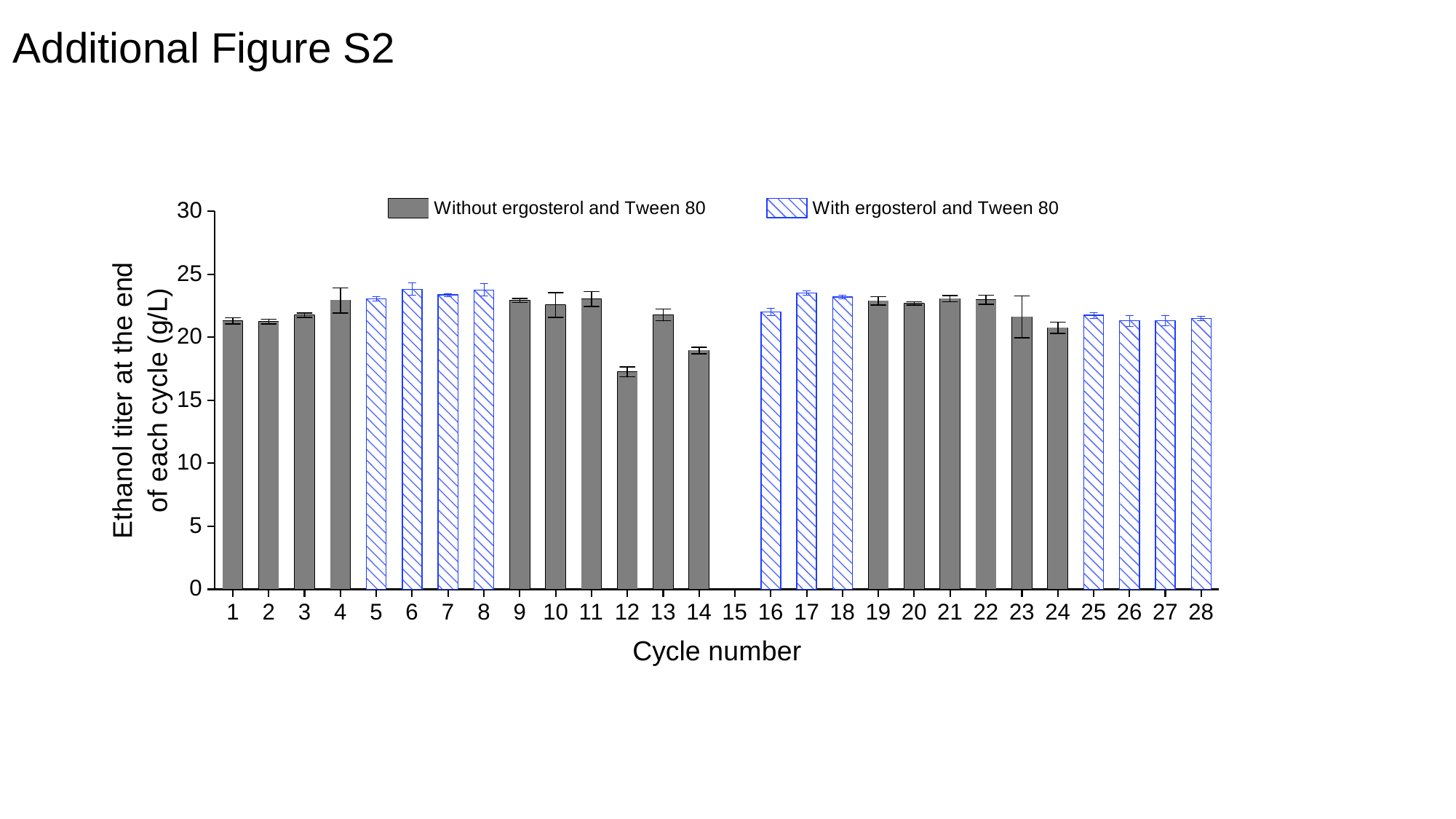

Additional Figure S2

Supplement: Supplementary file 2 — Additional file 2: Figure S2. Final ethanol titer at the end of each SCF cycle. SCF was performed using medium without or with the supplementation of ergosterol (0.02 g/L) and Tween 80 (0.8 g/L). The data reported is the average of analytical triplicates, with error bars representing standard deviations. No samples were collected for the end of cycle 15. [file 13068_2020_1658_MOESM2_ESM.pptx]

## Slide 1
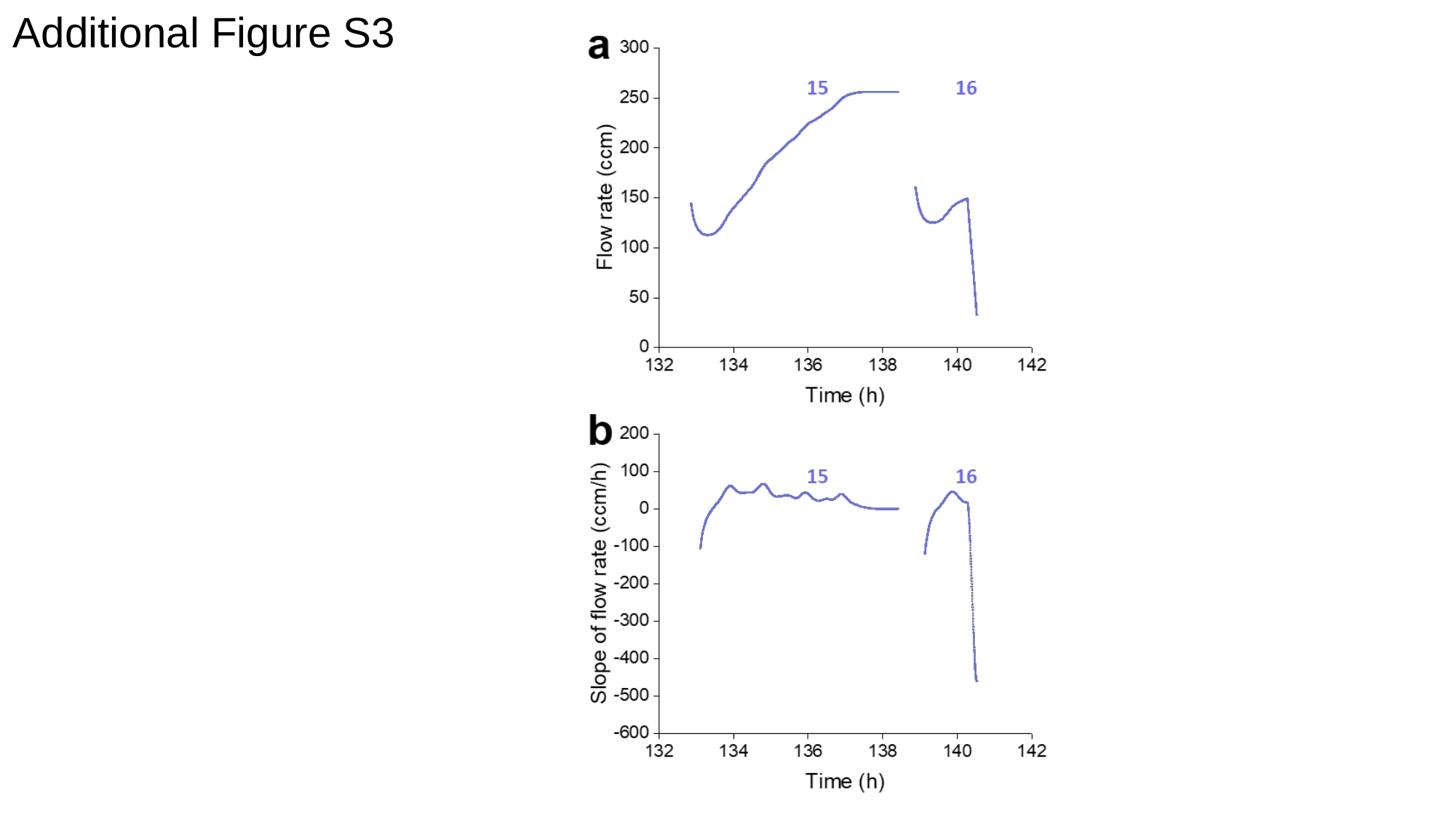

Additional Figure S3

Supplement: Supplementary file 3 — Additional file 3: Figure S3. Ergosterol and Tween 80 supplementation for cycle 15 and 16. Medium with supplementation of ergosterol (0.02 g/L) and Tween 80 (0.8 g/L) was used for these two cycles. Cycle numbers were labeled at the top of each figure. Cycle 15 and part of cycle 16 were removed from c and d, due to excessive flow of nitrogen to the fermenter as a result of nitrogen regulator failure; however, they are displayed here for gas flow rate (a) and slope of gas flow rate (b). [file 13068_2020_1658_MOESM3_ESM.pptx]

## Slide 1
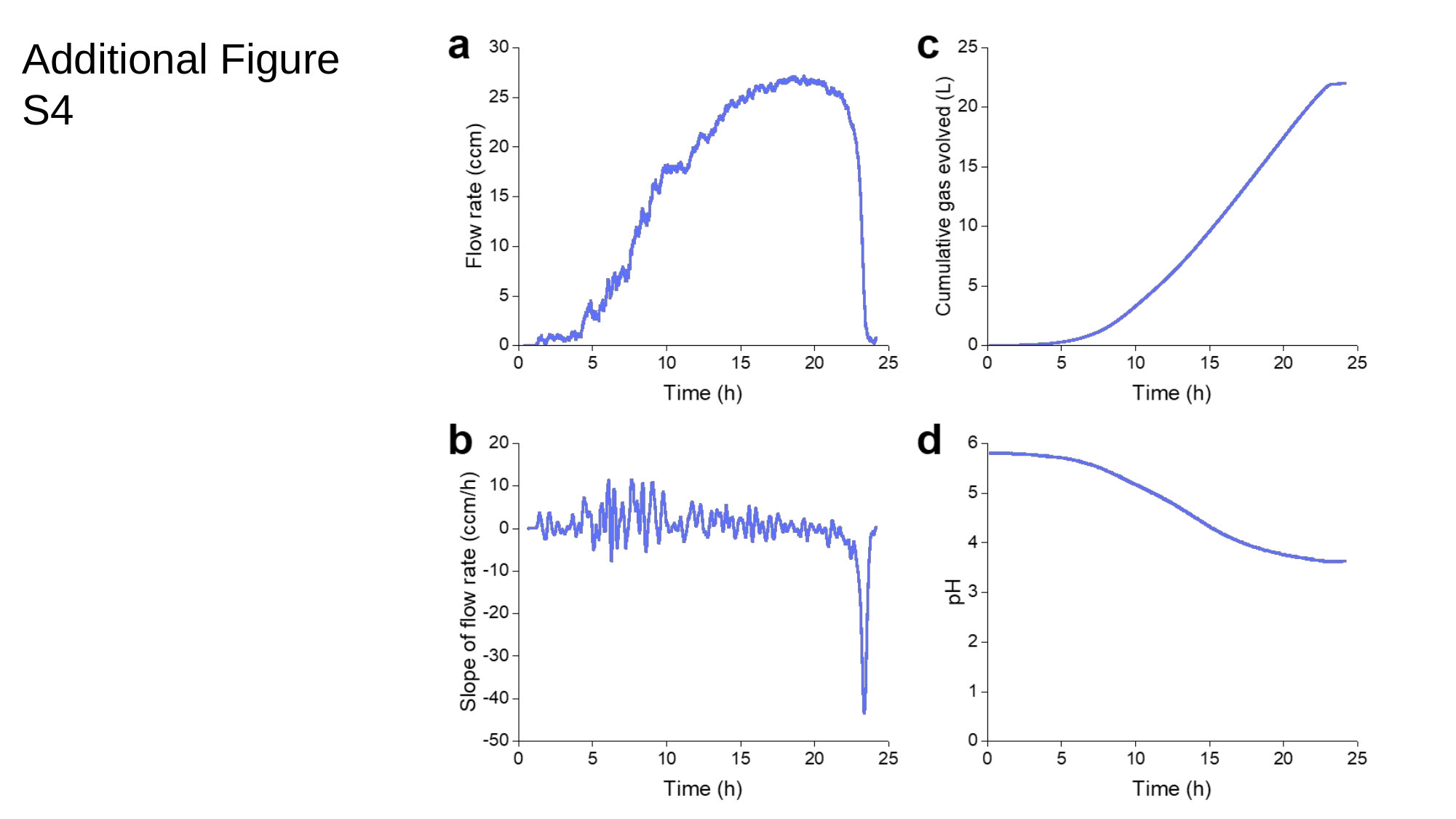

Additional Figure S4

Supplement: Supplementary file 5 — Additional file 5: Figure S4. Batch fermentation with supplements. Medium supplemented with ergosterol (0.02 g/L) and Tween 80 (0.8 g/L) was used. Gas flow rate (a), slope of gas flow rate (b), total gas captured per cycle (c), and pH (d) were monitored throughout SCF operation. [file 13068_2020_1658_MOESM5_ESM.pptx]

## Slide 1
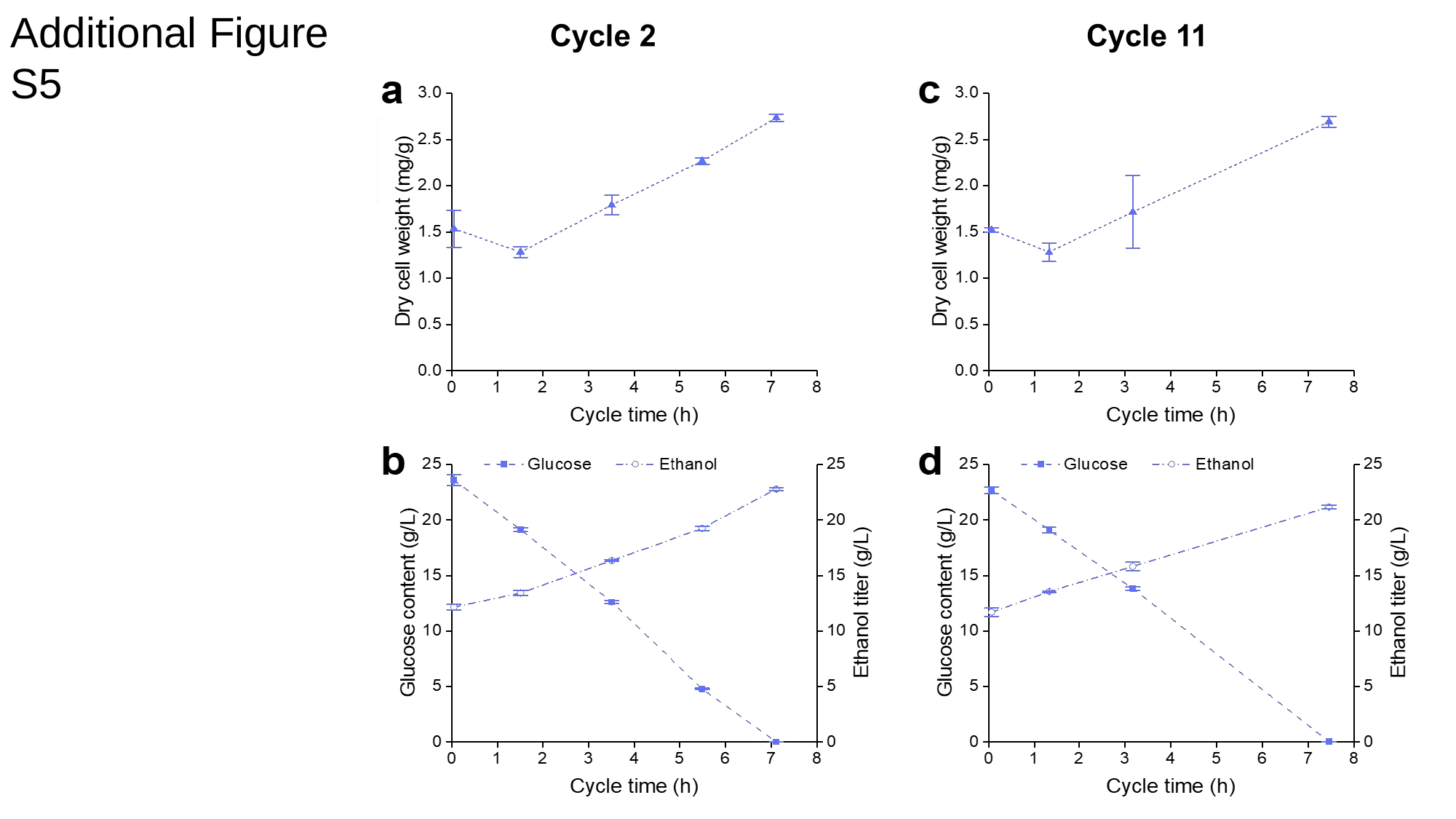

Additional Figure S5

Supplement: Supplementary file 6 — Additional file 6: Figure S5. Intracycle sampling during SCF demonstration. Biomass dry cell weight, glucose and ethanol concentrations were plotted for cycle 2 (a and b) and 11 (c and d). Medium supplemented with ergosterol (0.02 g/L) and Tween 80 (0.8 g/L) was used. The data reported is the average of analytical triplicates, with error bars representing standard deviations. [file 13068_2020_1658_MOESM6_ESM.pptx]

## Slide 1
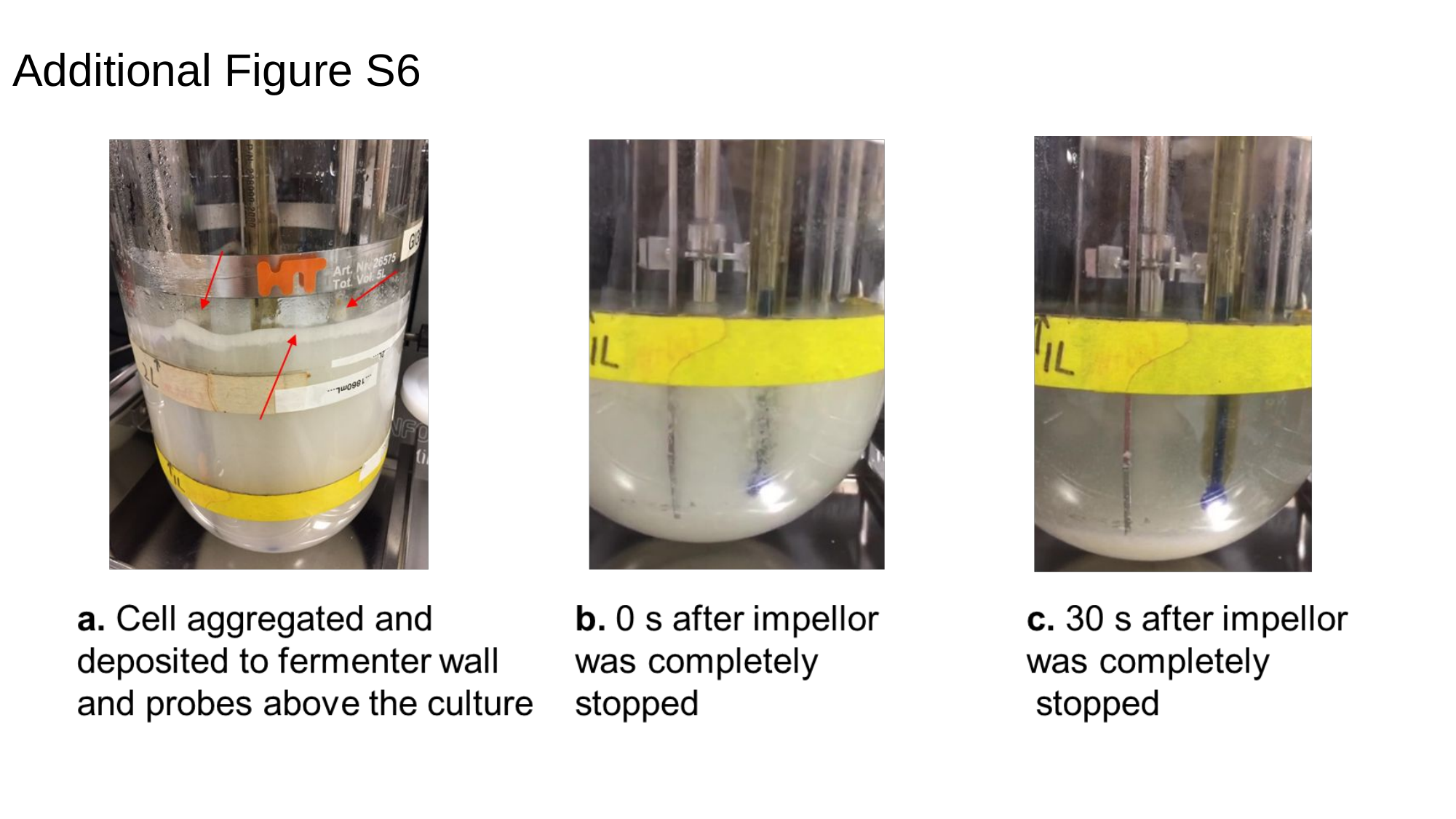

Additional Figure S6

Supplement: Supplementary file 7 — Additional file 7: Figure S6. Cell deposition and flocculation observed during SCF. A picture was taken at the end of cycle 21 (cycle time of 6.4 h) for a demonstration of cell deposition (a). Pictures were also taken at 0 s (b) and 30 s (c) after the impellors were completely stopped for cycle 21 to show the effect of flocculation. [file 13068_2020_1658_MOESM7_ESM.pptx]
