# Supplementary material for: Improved bioethanol productivity through gas flow rate-driven self-cycling fermentation
Source: Biotechnol Biofuels. 2020 Jan 24;13:14. doi: 10.1186/s13068-020-1658-6 (PMC6979077; doi:10.1186/s13068-020-1658-6)
Supplement: Supplementary file 4 — Additional file 4: Table S1. Comparison between batch and SCF operation under similar conditions. [file 13068_2020_1658_MOESM4_ESM.docx]

**Additional information**

**Additional Table S1.** Comparison between batch and SCF operation under similar conditions.

| Operation mode | Cycle time, (h) | Ethanol produced (g/L) | Fermentation efficiency  (%) | Annual ethanol productivity*  (ton/year) |
| --- | --- | --- | --- | --- |
| Batch | 23.4 | 21.5 ± 0.1 | 81.1 ± 0.8 | 580 ± 3 |
| SCF  Cycle 1  Cycle 4  Cycles 2,3,5-21 | 22.6  10.7  6.7-7.5 | 22.4 ± 0.5  10.5 ± 0.3  9.5-11.2 | 80.8-91.2 | 1020 ± 16 |

*Based on a fermenter of volume 10^5^ L.

Mean values from triplicate analysis ± standard deviation.
